# De novo *NSF* mutations cause early infantile epileptic encephalopathy

**DOI:** 10.1002/acn3.50917

**Published:** 2019-11-01

**Authors:** Hisato Suzuki, Takeshi Yoshida, Naoya Morisada, Tomoko Uehara, Kenjiro Kosaki, Katsunori Sato, Kohei Matsubara, Toshiyuki Takano‐Shimizu, Toshiki Takenouchi

**Affiliations:** ^1^ Center for Medical Genetics Keio University School of Medicine Tokyo Japan; ^2^ Department of Pediatrics Kyoto University Graduate School of Medicine Kyoto Japan; ^3^ Department of Clinical Genetics Hyogo Prefectural Kobe Children’s Hospital Hyogo Japan; ^4^ Applied Biology and Advanced Insect Research Promotion Center Kyoto Institute of Technology Kyoto Japan; ^5^ Department of Pediatrics Keio University School of Medicine Tokyo Japan

## Abstract

N‐ethylmaleimide‐sensitive factor (NSF) plays a critical role in intracellular vesicle transport, which is essential for neurotransmitter release. Herein, we, for the first time, document human monogenic disease phenotype of de novo pathogenic variants in *NSF*, that is, epileptic encephalopathy of early infantile onset. When expressed in the developing eye of *Drosophila*, the mutant *NSF* severely affected eye development, while the wild‐type allele had no detectable effect under the same conditions. Our findings suggest that the two pathogenic variants exert a dominant negative effect. De novo heterozygous mutations in the *NSF* gene cause early infantile epileptic encephalopathy.

## Introduction

Intracellular vesicle transport and endo/exocytosis are fundamental processes underlying a wide range of biological activities, including neurotransmitter release and hormone secretion. The delivery and release of cargo proteins are precisely regulated by a number of proteins.^1^ N‐ethylmaleimide‐sensitive factor (NSF) is a homo‐hexameric AAA ATPase that is involved in membrane fusion.^2^ The membrane receptors for NSF were identified and named as the soluble NSF attachment protein receptor (SNARE) complex.^3^ Assembly and disassembly of NSF and the SNARE complex, along with calcium triggering at appropriate location and time, are critical steps in vesicular transport and membrane fusion. The discovery and understanding of this trafficking machinery were awarded the Nobel Prize in Physiology or Medicine in 2013.^4^

Dysregulation of this intracellular vesicular transport/membrane fusion process has been increasingly recognized as the mechanistic basis for the development of diabetes and neurological disorders. Indeed, pathogenic variants of the SNARE complex are known to be associated with the development of Alzheimer's disease, Parkinsonism, autism, and psychiatric disorders.^5^ However, monogenic disease phenotype of *NSF* mutations has not been described previously. In invertebrates, the *Drosophila* homolog of *NSF,* that is,* comatose* (*comt*), was first mapped and cloned in association with temperature‐sensitive paralysis four decades ago.^6^, ^7^ The *comt* mutant flies show a burst of electrical discharges of the thoracic flight muscles at high temperatures,^8^ akin to febrile seizures in humans. Here, we describe two patients with de novo pathogenic variants in *NSF* with early infantile epileptic encephalopathy, to demonstrate that pathogenic variants of *NSF* cause the monogenic epileptic phenotype in humans.

## Materials and Methods

### Subjects and exome sequencing

The present research protocol was approved by the local ethics committees. Written informed consent was obtained from the parents for the analyses. As part of nationwide clinical projects, that is, Initiative on Rare and Undiagnosed Diseases (IRUD) and Rapid Genetic Diagnosis toward Neonatal Precision Medicine, conducted by Japan Agency for Medical Research and Development, exome analyses in trios were conducted in subjects with suspected genetic diseases who fulfilled the enrollment criteria.^9^ The enrollment criteria included the presence of multiple organ symptoms and history of familial inheritance. Since July 2015, more than 1300 undiagnosed families were enrolled in these projects. Genomic DNA was extracted from the peripheral blood leukocytes of patients 1 and 2 and their parents. Whole‐exome sequencing in patients and their parents was performed, as described previously.^10^

### Functional assay using the *Drosophila* model

To assess the functional relevance of the amino acid changes identified in the two patients, we introduced exogenous human *NSF* alleles carrying p.Ala459Thr and p.Pro563Leu to *Drosophila*. We ectopically expressed the wild‐type and two mutant *NSF* genes in the nervous system of the *Drosophila* using the GAL4/UAS system. A full‐length cDNA clone of the human *NSF* gene (clone 4812117) was obtained from DNAFORM (Yokohama, Japan). The entire coding sequence was amplified with and without a stop codon by PCR and subcloned into the pENTR221 vector using the In‐Fusion® HD Cloning Kit (Takara Bio USA, Inc, CA). The subclones were sequenced and confirmed to have the correct sequence. The two proband mutations (c.1375G>A, p.Ala459Thr and c.1688C>T, p.Pro563Leu) were introduced into the *NSF* coding sequence by site‐directed mutagenesis. Based on the Gateway technology, the cloned genes were transferred into the destination vectors, pUASg‐attB and pUASg‐HA_attB.^11^ The plasmid DNAs were injected into embryos carrying the *attP40* landing site for phiC31 integrase‐mediated transformation (*y* ^1^ *v* ^1^ *P{y[+t7.7]=nos‐phiC31\int.NLS}iedX*; *P{y[+t7.7]=CaryP}attP40*). Five virgin females of *GAL4* driver strains were crossed with five males of the *UAS‐NSF* strains and transferred every 3 days to a new vial. In order to detect dying cells in the developing eye discs, a TUNEL (terminal deoxynucleotidyl transferase‐mediated dUTP nick end‐labeling) assay was performed.^12^ The host strain for transformation and the *nSyb‐GAL4* strains (*y^1^ w^1118^; P{y[+t7.7] w[+mC]=nSyb‐GAL4.P}attP2* and y^1^ w; P{w[+m*]=nSyb‐GAL4.S}3) were obtained from Bloomington Drosophila Stock Center and the *P{GAL4‐ninaE.GMR}* driver strain (*w P{w[+mC]=GAL4‐ninaE.GMR}12*) was procured from the KYOTO Stock Center. These flies carried intact intrinsic *comt/NSF1* and *NSF2*.

## Results

### Clinical description

**Figure 1:**
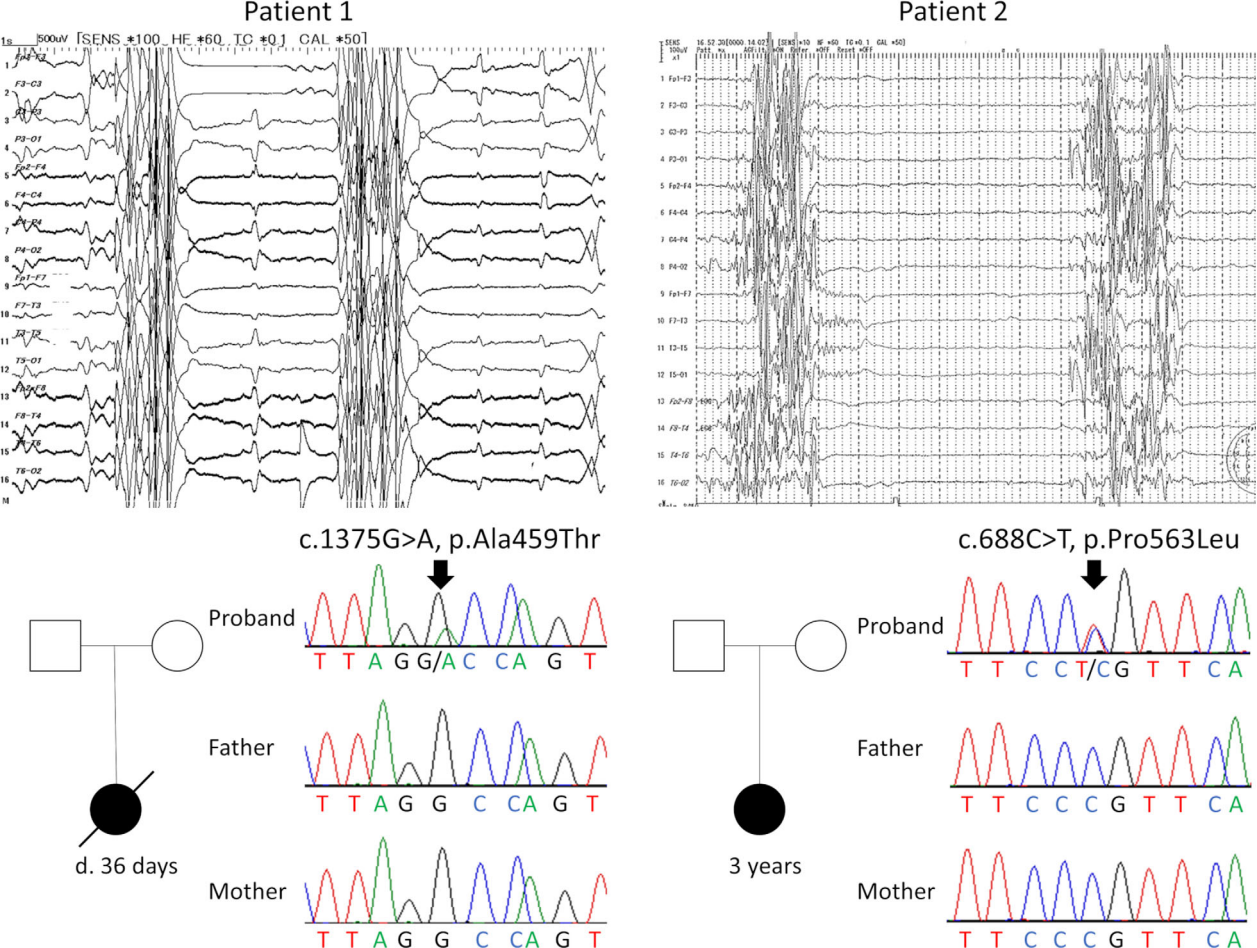
Clinical characteristics of the two patients. Top: The electroencephalographs of both patients 1 and 2 showed a continuous burst‐suppression pattern, regardless of sleep–wake stage. The burst phase lasted for a duration of 2–5 sec and consisted of diffuse irregular spikes and sharp waves measuring 100–250 *µ*V in amplitude. The suppression phase lasted for 5–20 sec and was nearly isoelectric. Bottom: Family pedigree analysis of patients 1 and 2 showed that no other family members were affected. The chromatograms of patient 1 showed c.1375G>A p.Ala459Thr in *NSF* in a heterozygous state (an arrow). Neither of the parents carried this pathogenic variant. The chromatograms of patient 2 showed c.1688C>T p.Pro563Leu in *NSF* in a heterozygous state (an arrow). Neither of the parents carried this pathogenic variant.

Patient 1: The first proband was a Japanese girl. She was born at 37 weeks of gestation by uneventful delivery after an uneventful pregnancy. There was no significant family medical history. Her birth weight was 2,930 g (+0.42 SD) and her head circumference was 33.5 cm (+0.38 SD). Immediately after birth, she developed continuous vomiting and tonic seizures. Electroencephalography on the ninth day after birth showed a continuous burst‐suppression pattern, regardless of the sleep–wake stage. The burst phase lasted for a duration of 2–5 sec and consisted of diffuse irregular spikes and sharp waves measuring 100–250 *µ*V in amplitude. The suppression phase lasted for 5–20 sec and was nearly isoelectric (Fig. 1). Based on the findings, this female infant was diagnosed as having early infantile epileptic encephalopathy. Despite maximum medical support in the pediatric intensive care unit, the infant died on day 36 after birth of respiratory failure.

Patient 2: The second proband was a 1‐year‐old Japanese girl with no significant family medical history. In her prenatal period, she exhibited failure to thrive, hydrops, and anemia, for which blood transfusion *via* the placenta was performed. She was born at 33 weeks of gestation *via* emergent cesarean section due to fetal distress. Her birth weight was 1,334 g (−2.1 SD) and her head circumference was 23.5 cm (−3.4 SD). After birth, she had no spontaneous respiration, which needed mechanical ventilation, and had frequent myoclonic seizures. Her electroencephalography showed continuous burst‐suppression patterns highly similar to that in patient 1 (Fig. 1). At the age of 3 years, she had profound intellectual disability, severe motor developmental delay, and no spontaneous respiration. Her electroencephalogram persistently showed burst‐suppression patterns. She continued to have frequent myoclonic seizures and epileptic spasms.

### Molecular analysis

**Figure 2:**
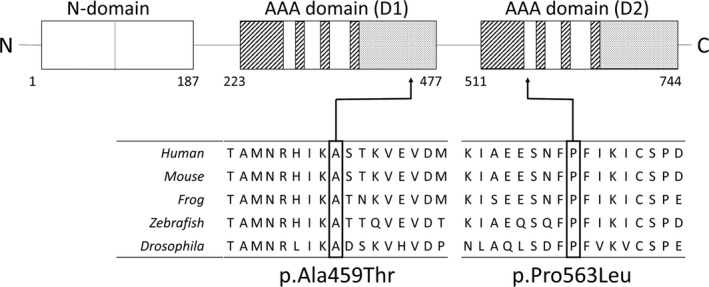
Top: A schematic representation of the NSF molecule. Ala459 is located in the AAA domain (D1). Pro563 is located in the AAA domain (D2). Bottom: Amino acid alignments at and around Ala459 and Pro563. Note that Ala459 and Pro563 are highly conserved among species.

Whole‐exome sequencing in the patients and their parents revealed a de novo heterozygous variants in the *NSF* gene (NM_006178.3) in both probands: c.1375G>A chr17:44782125G>A (GRCh37) p.Ala459Thr in patient 1 and c.1688C>T chr17:44791279C>T (GRCh37) p.Pro563Leu in patient 2. The results were confirmed by Sanger sequencing. Neither of the two variants were present in the exome datasets of more than 60,000 individuals without severe pediatric diseases (the ExAC database (http://exac.broadinstitute.org/)) or in a cohort of 2049 Japanese normal individuals (Integrative Japanese Genome Variation Database (https://ijgvd.megabank.tohoku.ac.jp/)). The codons Ala459 and Pro563 in the *NSF* gene are highly conserved among many species (Fig. 2). No candidate genes were identified in the autosomal recessive model.

In silico analyses showed that the combined annotation‐dependent depletion score, which reflects the relative pathogenicity of human variants,^13^ was 30 for p.Ala459Thr, and 34 for p.Pro563Leu. According to the American College of Medical Genetics guideline for the pathogenicity of variants,^14^ the two variants were classified as “pathogenic.”

### Functional analysis of the pathogenic variants using *Drosophila.*

**Figure 3:**
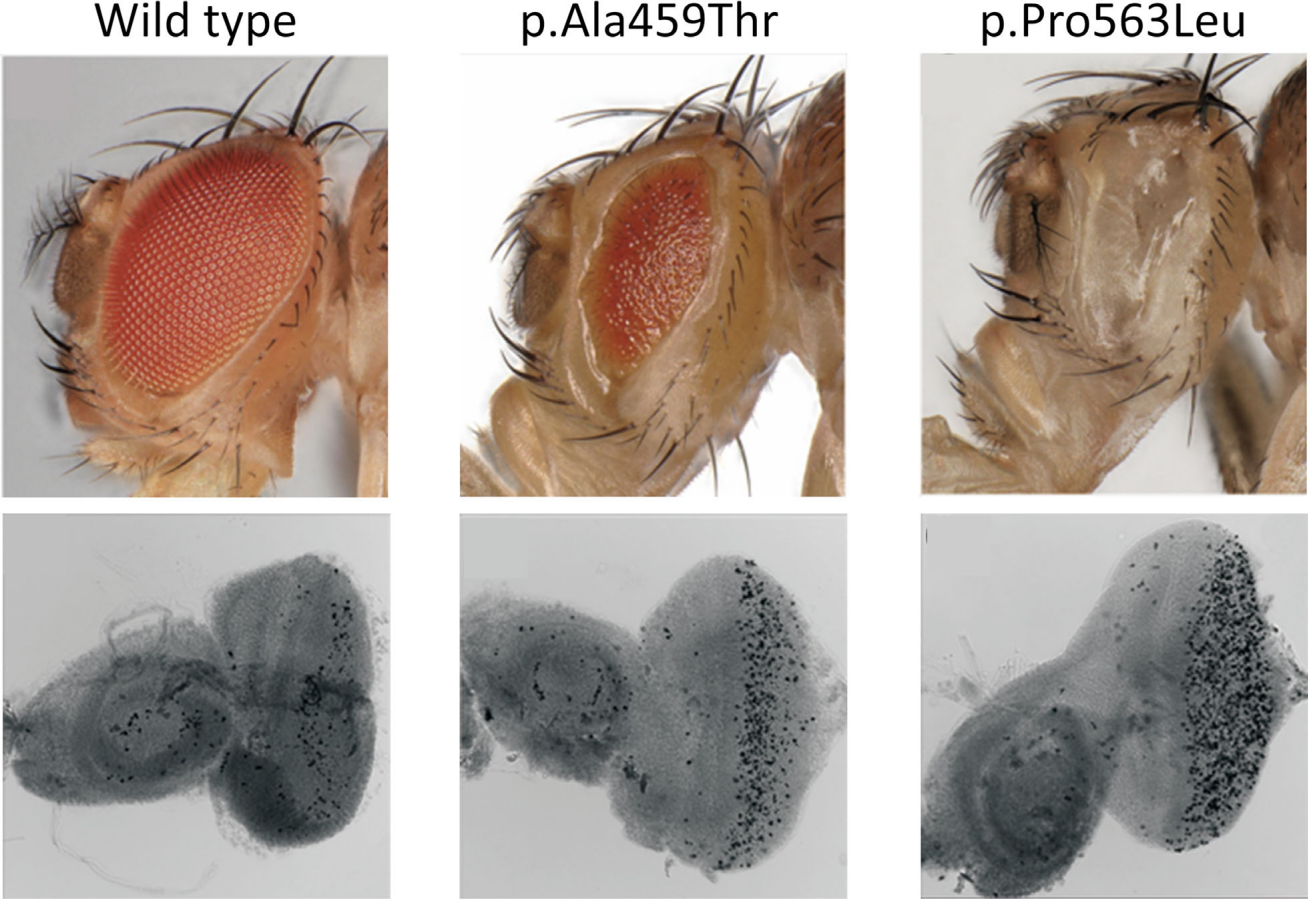
Functional effects of the two *NSF* pathogenic variants on the eye discs of *Drosophila*. Top: Photographs of the eyes of *Drosophila* females expressing the *NSF* gene under the control of the *P{GAL4‐ninaE.GMR}* driver. Note that the mutants, but not the wild‐type *NSF* genes, led to defective eye phenotypes when expressed in the developing eyes. Left: *P{GAL4‐ninaE.GMR}*/*UAS‐NSF* (the wild‐type allele); Middle: *P{GAL4‐ninaE.GMR}*/*UAS‐NSF^A459T^*; Right: *P{GAL4‐ninaE.GMR}*/*UAS‐NSF^P563L^*. Bottom: TUNEL images of the eye imaginal discs of larvae expressing the *NSF* gene under the control of the *P{GAL4‐ninaE.GMR}* driver. Left: *P{GAL4‐ninaE.GMR}*/*UAS‐NSF* (wild‐type allele); Middle: *P{GAL4‐ninaE.GMR}*/*UAS‐NSF^A459T^*; Right: *P{GAL4‐ninaE.GMR}*/*UAS‐NSF^P563L^*. TUNEL‐positive cells are seen in black and also in white (out‐of‐focus). Note that TUNEL‐positive dying cells are more abundant in the developing eye discs expressing the mutant *NSF* genes than in those expressing the wild‐type *NSF* gene.

Although the wild‐type *UAS‐NSF* gene combined with the *GAL4* driver gene did not result in any detectable phenotype, both flies carrying the mutant *UAS‐NSF* and *P{GAL4‐ninaE.GMR}* genes exhibited defective eyes. Especially, the *P{GAL4‐ninaE.GMR}/UAS‐NSF^P563L^* flies exhibited complete obliteration of the eyes, while massive cell death was observed in the developing eye discs (Fig. 3). Ectopic expression of the mutated *NSF* genes under the control of the pan‐neuronal *nSyb‐GAL4* driver genes resulted in embryonic or the first instar larval lethality (data not shown).

## Discussion

In the present report, we, for the first time, document epileptic encephalopathy of infantile onset caused by de novo heterozygous pathogenic variants in the gene encoding NSF, a molecule that is critical for intracellular vesicle transport and membrane fusion. To analyze the functional relevance of the human *NSF* pathogenic variants observed in the patients, the human mutant alleles were overexpressed in the *Drosophila* eye discs using the *GAL4‐UAS* system. The flies expressing the human mutant *NSF* alleles in the developing eye discs exhibited defective eye development, suggesting that the pathogenic variants exerted a dominant negative effect. Collectively, we herein establish that pathogenic variants in the *NSF* cause early infantile epileptic encephalopathy.

Functional analysis of mutant *Drosophila* is a well‐established method in the evaluation of pathogenic variants.^15^ In the *Drosophila* model, overexpression of a loss‐of‐function allele results in no overt phenotype, whereas overexpression of gain‐of‐function alleles results in overt phenotypes.^16^ In the present study, while overexpression of the two mutant alleles resulted in defective eye development, overexpression of the wild‐type allele had no such effect. These observations suggest that the amino acid substitution changes (p.Ala459Thr and p.Pro563Leu) in the two patients exerted a dominant negative effect. Yet, it still remains to be explored how mutant proteins exert a dominant negative effect. NSF forms a homomeric hexamer.^17^ Given the similarity of the amino acid alignment between exogenous *NSF* alleles and intrinsic *comt/NSF* alleles, the proteins arising from exogenous mutant alleles likely interfere with the intrinsic NSF product during the formation of multimers.

Our animal model had intact intrinsic *comt/NSF1* and *NSF2*. We introduced human mutant alleles using the *GAL4‐UAS* driver to express human mutant alleles exclusively in the *Drosophila* eye discs. Generation and functional analysis of a transgenic model may provide further insight into the pathogenetic mechanism arising from the two *NSF* mutations that were identified in the patients. Nonetheless, the observation that both flies with abnormal function in *comt* and infants with abnormal function in *NSF* exhibited epileptic phenotype illustrates that two orthologs (*comt* and *NSF*) in the two different species are related to the abnormal electrical discharges and epilepsy phenotype.

Pathogenic variants in proteins involved in the regulation of intracellular vesicular transport and membrane fusion are known to cause epileptic encephalopathies. STXBP1 (MUNC18‐1) and NECAP1 are regulatory proteins that are involved in synaptic neurotransmitters release. Pathogenic variants in these genes are known to cause epileptic encephalopathy of early infantile onset.^18^, ^19^ Considering that NSF is involved in the intracellular vesicle transport and recycling of vesicles^20^ and the development of central nervous system malformation in the mutant *Drosophila*, the epileptic phenotype observed in the two reported patients is likely caused by dysregulation of synaptic neurotransmission.

Besides playing roles in intracellular vesicle transport and synaptic transmission of neurotransmitters, neurogenesis, and neuroprotection, NSF may also play a role in the epileptic phenotype. *Drosophila* carrying mutant *NSF/comt* exhibited a reduced life‐span and progressive neurodegeneration of the dopaminergic neurons, suggesting involvement of *NSF* in autophagy and neuroprotection.^21^ Moreover, the lethal mutation in *comt* resulted in a reduction in the size and branching of synapses, suggesting that *NSF* plays a critical role in neurogenesis.^22^ In our Drosophila model, ectopic expression of mutant *NSF* (i.e*.*, p.Ala459Thr and p.Pro563Leu) under the control of the pan‐neuronal nSyb‐GAL4 driver showed embryonic or first instar larval lethality, and the finding of TUNEL‐positive dying cells observed in the developing eye discs appeared to be consistent with the critical roles of *NSF* in neurogenesis and perhaps in neuroprotection. Although the paralytic phenotype in flies does not prove but suggests that *NSF* dysfunction causes the epilepsy phenotype, we could not determine which mechanisms, that is, neurogenesis and/or neuroprotection, of the *NSF* are relevant to the patients' epileptic phenotype. Further studies employing anatomical and physiological analyses are needed.

From a clinical standpoint, defining the exact phenotypic spectrum of this presumably new early infantile epileptic encephalopathy would have implications in the clinical management. Both patients exhibited frequent myoclonic seizures accompanied by burst‐suppression patterns on electroencephalogram within 1 week after birth. These clinical features during the neonatal period were essentially nondistinguishable from those in early infantile epileptic encephalopathy due to *KCNQ2* and *SCN2A* mutations. However, it was notable that the electroencephalogram in patient 2 showed persistent burst‐suppression patterns even beyond 3 years of age, which has not so far been described in cases of early infantile epileptic encephalopathy caused by *KCNQ2* or *SCN2A* mutations.^23^ Further investigations in a larger cohort of patients are warranted to explore characteristic electroencephalographic progression and optimal antiepileptic strategies for patients with *NSF*‐related early infantile epileptic encephalopathy.

In summary, two unrelated infants carrying de novo heterozygous pathogenic variants in the *NSF*, that is*,* p.Ala459Thr and p.Pro563Leu, presented with epileptic encephalopathy of early infantile onset. The functional analysis using the *Drosophila* eye disc model suggested the pathogenicity of the variants, that is, that they exerted dominant negative effects. We conclude that de novo heterozygous mutations in the *NSF* cause early infantile epileptic encephalopathy.

## Conflict of Interest

None of the authors have any conflict of interest to disclose.
